# How the Tumor Micromilieu Modulates the Recruitment and Activation of Colorectal Cancer-Infiltrating Lymphocytes

**DOI:** 10.3390/biomedicines10112940

**Published:** 2022-11-15

**Authors:** Imke Atreya, Markus F. Neurath

**Affiliations:** 1 Department of Medicine 1, Erlangen University Hospital, Friedrich-Alexander-University Erlangen-Nürnberg, 91054 Erlangen, Germany; 2 Deutsches Zentrum Immuntherapie (DZI), Erlangen University Hospital, Friedrich-Alexander-University Erlangen-Nürnberg, 91054 Erlangen, Germany

**Keywords:** colorectal cancer, tumor-infiltrating lymphocytes, chemokines, cytokines

## Abstract

The successful treatment of advanced colorectal cancer disease still represents an insufficiently solved clinical challenge, which is further complicated by the fact that the majority of malignant colon tumors show only relatively low immunogenicity and therefore have only limited responsiveness to immunotherapeutic approaches, such as, for instance, the use of checkpoint inhibitors. As it has been well established over the past two decades that the local tumor microenvironment and, in particular, the quantity, quality, and activation status of intratumoral immune cells critically influence the clinical prognosis of patients diagnosed with colorectal cancer and their individual benefits from immunotherapy, the enhancement of the intratumoral accumulation of cytolytic effector T lymphocytes and other cellular mediators of the antitumor immune response has emerged as a targeted objective. For the future identification and clinical validation of novel therapeutic target structures, it will thus be essential to further decipher the molecular mechanisms and cellular interactions in the intestinal tumor microenvironment, which are crucially involved in immune cell recruitment and activation. In this context, our review article aims at providing an overview of the key chemokines and cytokines whose presence in the tumor micromilieu relevantly modulates the numeric composition and antitumor capacity of tumor-infiltrating lymphocytes.

## 1. Introduction

Colorectal cancer (CRC) represents the third most common malignant tumor disease worldwide and was found to be the underlying diagnosis in almost 10% of all cancer-related deaths in 2020 [1]. In the subgroup of CRC patients who have already developed distant metastases (stage IV disease), the average 5-year relative survival rate is only 12% [2]. Therefore, significant scientific efforts are being made to gain a more detailed understanding of the CRC-specific pathogenesis and to translate this into clinically applicable strategies for improved early diagnosis and optimized therapy [3].

It is well accepted that the tumor microenvironment and, in particular, the local presence of immune and stromal cells critically determine CRC prognosis [4]. Already in 2005, in a pioneering study published by the team of Jérôme Galon, it was demonstrated that the accumulation of early memory and effector memory CD8^+^ T cells in the tumors of CRC patients correlated significantly with an improved clinical outcome (absence of early metastatic invasion, a less advanced pathological stage, and increased survival) [5]. Meanwhile, this recognized relevance of the tumors’ immune profile for CRC prognosis has been successfully translated into clinically applied diagnostic concepts. For instance, the Immunoscore, a standardized immune assay based on the immunohistochemical staining of tumor-infiltrating CD3^+^ and CD8^+^ T cells in combination with automated artificial intelligence-assisted signal detection and interpretation, has found its way into international consensus guidelines for the clinical management of CRC disease and has been proven to refine prognosis and significantly improve CRC risk assessment [6,7]. 

However, although the development of CRC usually results in the induction of profound antitumor immune responses indicated, for instance, by the presence of tumor antigen-specific adaptive immune cells in the peripheral blood of affected patients [8], the local tumor microenvironment often succeeds in generating local tumor-beneficial conditions, which are able to dampen the accumulation and cytotoxic activity of infiltrating effector T cells and to promote the immune escape properties of tumor cells [9]. As a prime example, DNA mismatch repair-proficient or microsatellite-stable CRC colon cancer, which represent around 85% of all diagnosed CRC diseases, are characterized by low overall immunogenicity and the decreased infiltration of CD8^+^ T lymphocytes [10,11,12]. The phenomenon of tumor immune escape includes, for instance, the enhanced infiltration of myeloid-derived suppressor cells and suppression-competent FoxP3^high^ regulatory T cells (T_reg_ cells), which results in suppressed effector T cell function and was described to be associated with CRC progression, lymphatic invasion, and metastasis [13,14]. As we will consider T_reg_ cells in the following as an antitumor immune response-suppressing and thus colon tumor growth-promoting T cell subpopulation, it is important to mention, at least briefly, the functional heterogeneity of FoxP3^+^ T cells in the CRC microenvironment [15]. While CRC-infiltrating FoxP3^high^ CD45RA^−^ T cells are characterized by a good capacity to regulate and suppress effector T cell function, FoxP3^low^ CD45RA^−^ T cells turned out to be non-suppressive but produced pro-inflammatory cytokines [15]. The intratumoral co-existence of suppression-competent FoxP3^high^ T_reg_ cells and FoxP3^low^ non-T_reg_ cells might most likely be responsible for the partly controversial discussion about the prognostic role of CRC-infiltrating FoxP3^+^ T cells [16,17].

Understanding that the fate of tumor disease is significantly influenced by the efficiency of the antitumor immune response and the overcoming of immune-evasive local conditions has also shaped the development of modern therapeutic strategies for the treatment of CRC patients. While classic chemotherapeutic treatment regimens predominantly target the proliferation and survival of tumor cells directly, more recent immunotherapeutic approaches instead aim at supporting anticancer immunity by modulating the tumor microenvironment. Thus, the principle of immunotherapy is primarily to increase the number of tumor-infiltrating and tumor antigen-specific cytotoxic T lymphocytes (e.g., by adaptive cell transfer [18,19] or by optimized recruitment of systemic effector cells) and to increase their cytolytic activity (e.g., by checkpoint inhibitors [11] or by inhibition of immunosuppressive components). However, a large proportion of CRC patients show only a rather disappointing response to monotherapy with clinically established PD-1 checkpoint inhibitors [11]. Thus, it currently remains an important challenge to better understand CRC-specific aspects in the interaction between tumor cells and their local microenvironment (including accumulating immune cells) in order to develop innovative immunotherapeutic strategies to optimize the efficacy of the immune system in controlling intestinal tumor growth. 

## 2. Prognostic-Relevant Chemokine Signaling Impacting the Immune System-Mediated Control of CRC

**Figure 1:**
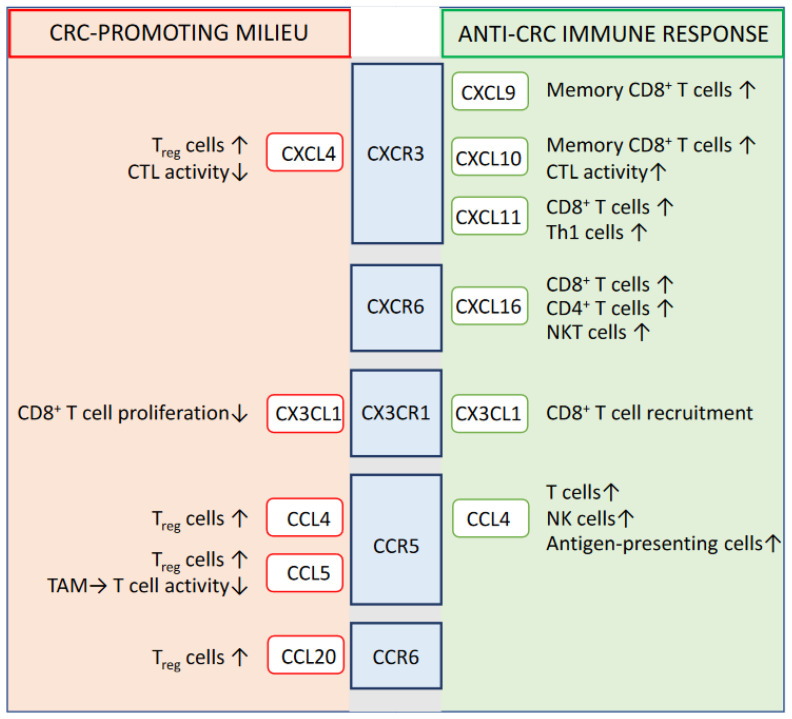
Chemokine signaling of described relevance for the immune system-mediated control of CRC. Abbreviations: TAM = tumor-associated macrophages; CTL = cytotoxic T lymphocytes; ↑ = increased; ↓ = decreased.

The demonstrated relevance of tumor-infiltrating lymphocytes to the fate of CRC disease has turned our attention also to those mediators in the close tumor environment that, through their chemotactic capacity, underlie the local accumulation of T cells and are able to influence the ratio between tumor-suppressing T lymphocytes and tumor-promoting T_reg_ cells. Several chemokines (e.g., CCL1, CCL3, CCL4, CCL7, CCL20, CCL25, CCL26, CX3CL1, CXCL1, CXCL8, CXCL9, CXCL10, and CXCL16) and partly also their respective G protein-coupled receptors (e.g., CCR1, CCR5, CCR6, CCR8, CXCR2, CXCR6, and CX3CR1) were found to be upregulated or selectively expressed in human colon cancer tissue compared to corresponding non-tumor control tissue [20,21,22,23,24,25]. Moreover, during the last two decades, significant progress has been achieved in defining specific chemokines or chemokine receptors whose intratumoral expression profile might allow a prediction of tumor prognosis in individual CRC patients. For instance, high expression levels of CXCR3, CXCR4, and CCL20 could be identified as a predictor of poor prognosis [26,27,28,29], while increased levels of CXCL16, CCL4, CX3CL1, CXCR6, CX3CR1, and CCR5 in CRC lesions turned out to indicate prolonged disease-free survival and were mostly associated with the profound accumulation of effector T cells in the tumor [4,21,30,31,32,33] (Figure 1). 

### 2.1. CXCR3

CXCL9, CXCL10, and CXCL11 are ligands of the chemokine receptor CXCR3, which is preferentially expressed by CD4^+^ and CD8^+^ T cells, but could also be detected on the surfaces of cancer cells [34]. In tumors of individual CRC patients, a strong degree of co-expression has been described for all three of these CXCR3 ligands, whose gene transcription in tumor cells and tumor-associated stromal cells (e.g., endothelial cells and fibroblasts) can obviously be triggered by the presence of interferons and the pro-inflammatory cytokine TNFα [31]. The enhanced intratumoral expression of CXCL11, as the presumably most potent CXCR3 agonist [34], turned out to correlate with the increased presence of CD4^+^ T helper (Th) cells and CD8^+^ cytotoxic T cells in human CRC lesions. Regarding the impact of CXCL11 on the different Th cell subtypes, its presence in CRC did not correlate with the accumulation of FoxP3^+^ T_reg_ cells and additionally performed transcriptional analyses implicated its particular relevance for T-bet-expressing Th1 cells, strongly pointing to a supportive role of CXCR3 engagement during the adaptive immune system-mediated tumor control [31]. This is also in accordance with another study describing the particular relevance of CXCL9 and CXCL10 for the intratumoral enrichment of memory CD8^+^ T cells and the finding that CXL10 secretion in colon cancer tissue was found to correlate with the frequency of granzyme B-expressing CD8^+^ tumor-infiltrating lymphocytes [30,35]. However, in contrast to its ligands, CXCL9, CXCL10, and CXCL11, the protein expression of the CXCR3 chemokine receptor protein in colon cancer tissue could be associated with increased tumor size, the occurrence of lymph node or distant metastasis, and poor survival [28]. Most likely, the implicated tumor-promoting effects of CXCR3 can be explained by the tumor cell autonomous expression of this chemokine receptor and its described positive impact on cancer cell motility, resulting in an increased metastasis-forming capacity [36,37]. In this context, CXCL4, as another CXCR3 ligand, might be of significant relevance. Indeed, increased expression of CXCL4 in tumor tissue indicated a decreased survival time in CRC patients [38], and experimental in vivo CRC models described the capacity of this chemokine to promote the proliferation and activation of T_reg_ cells, while dampening CD8^+^ T cell-mediated antitumor immune responses [39]. Thus, it becomes crucial to consider and further elucidate the, at least partially, ambiguous and target cell-specific role of CXCR3 signaling [36,40] when discussing this chemokine receptor and its ligands as potential targets for CRC immunotherapy. 

### 2.2. CXCR6

Unlike this complex role of CXCR3 signaling in the context of CRC disease, which apparently can directly influence tumor cell behavior in addition to immune cell recruitment, chemokine receptors with a more restricted expression pattern limited to specific types of immune cells may represent more favorable therapeutic targets, in the sense that their effect on the fate of tumor development can be more precisely predicted. For instance, the chemokine receptor CXCR6 is expressed almost selectively by CD4^+^ T cells, CD8^+^ T cells, NKT cells, NK cells, plasma cells [41], while no or only very weak CXCR6 protein expression could be detected in colon cancer cells and, of particular relevance, its increased expression and the enhanced presence of its unique ligand CXCL16 in colon tumor tissue turned out to be associated with marked T cell infiltration and improved tumor prognosis [25,32,42]. Although human colon cancer tissue characterized by high expression levels of CXCL16 showed increased infiltration by CD4^+^ and CD8^+^ lymphocytes, data acquired in an experimental in vivo model of CRC metastasis interestingly indicated the particular relevance of the CXCL16/CXCR6-driven NKT cell recruitment for the control of hepatic CRC metastasis, while the beneficial effect of CXCL16 in this context seemed to be mostly independent of the presence or absence of CD8^+^ T cells [42]. However, CXCR6 expression can obviously be induced on the surface of colon tumor-infiltrating CD8^+^ T cells by yet-to-be-defined mediators of the local microenvironment, and CXCR6^+^ CD8^+^ T cells in primary human CRC lesions could recently be characterized by the marked expression of cytotoxic effector markers, indicating their potent antitumor effector function [25]. Indeed, the absence of CXCR6 in the transplantable MC38 colon tumor model drastically reduced the response to immune checkpoint therapy and decreased the number and cytotoxic effector activity of tumor-infiltrating CD8^+^ T cells [25]. Thus, the ex vivo selection or enrichment of CXCR3^+^ CD8^+^ T cells appears as a promising strategy for optimizing the efficacy of therapeutic concepts, which are based on the adoptive transfer of tumor-targeting T cells in the clinical context of CRC [25]. 

### 2.3. CX3CR1

Regarding CX3CL1 and its receptor CX3CR1, recent data interestingly indicated that antigen-specific tumor-infiltrating CD8^+^ T cells expressing high levels of CX3CR1 show a lower proliferative capacity upon antigenic rechallenge than their CX3CR1-negative counterparts and might be less relevant for the efficient immunological control of tumor growth [43]. However, considering the mentioned positive association between CX3CL1 and tumor prognosis [30,44], as well as its inverse correlation with lymph node metastasis [23] and the fact that the absence or decreased expression of the CX3CR receptor itself was found to be associated with the development of lymph node metastasis and poor CRC prognosis [4], it can be concluded that this chemokine/chemokine receptor interaction relevantly influences the fate of CRC disease by means other than triggering the antitumor activity of recruited immune cells [23]. Indeed, however, taking advantage of the fact that CX3CL1 seems to be present in more than 80% of colorectal tumors but showed a significantly lower expression level in the normal colon mucosa [22,23], ectopic expression of its receptor CX3CR on ex-vivo-expanded T cells designated for adoptive T cell immunotherapy has been discussed and successfully validated in vivo in a humanized CRC mouse model as a strategy to guide the transferred T cells from the peripheral circulation into the tumor tissue [22].

### 2.4. CCR5

As another chemokine typically expressed in the intestinal mucosa and usually upregulated in colorectal cancer tissue, CCL4 (previously known as macrophage inflammatory protein (MIP)-1β) is mainly produced by hematopoietic cells and, besides mediating the chemoattraction of macrophages and NK cells, also promotes the intestinal recruitment of T cells via its receptor CCR5 [45,46,47]. In addition to tumor-infiltrating immune cells, colon tumor cells might represent a CRC-intrinsic and β-catenin-regulated source of CCL4 [48], as suggested in analogy to a similar phenomenon observed in the context of cutaneous melanoma disease [49]. Although experimental in vivo administration of recombinant CCL4 failed to promote the cytolytic activation of mucosal CD8^+^ T cells and preferentially triggered Th2 cytokine responses [50], the intratumoral release of CCL4 can be expected to promote the recruitment of CD103^+^ dendritic cells and to subsequently enhance T cell priming against tumor-associated antigens [49]. Accordingly, increased protein expression levels of the CCL4-regulating protein β-catenin and low mRNA levels of CCR5 in CRC tissue turned out to be associated with decreased numbers of tumor-infiltrating CD8^+^ T cells [33,48] and, moreover, low CCL4 protein expression levels in colonic tumor tissue were associated with a significantly decreased cancer-specific survival rate in CRC patients [21]. Despite this obviously beneficial role of CCL4 for the immunological control of CRC disease, it cannot be neglected that colon tumor-infiltrating regulatory T cells (T_reg_ cells) could be characterized by significantly increased expression of the CCL4 receptor CCR5 compared to tumor-infiltrating conventional T cells but also in comparison to T_reg_ cells located in the mucosa of the distal colon [47]. As the targeted migration of CRC patient-derived T_reg_ cells towards CCL4 could be demonstrated in vitro [47], it thus appears likely that CCL4 can also support the T_reg_ cell-mediated immune evasion of colorectal tumors. Indeed, in the CT26 mouse colon cancer model, which is based on the subcutaneous injection of colon tumor cells, CCR5-deficient mice could be characterized by significantly decreased numbers of tumor-infiltrating T_reg_ cells and reduced tumor growth [51]. In the human system, the intratumoral protein expression of CCR5 markedly varies between individual CRC patients, being absent or only weakly expressed in almost 50% or 30% of patients, respectively. In accordance with the previously described beneficial role of the CCR5 ligand CCL4 in the immunological control of colonic tumorigenesis, CRC tumors with intermediate to strong CCR5 expression seemed to have a better prognosis, indicated by Union International Contre Cancer (UICC) staging and less frequent lymph node involvement [33]. However, CCL4 is not the only ligand of CCR5. In particular, CCL3 and CCL5 should also be considered as potential modulators of antitumor immune responses in the context of CRC, with CCL3 being significantly upregulated on the mRNA and protein level in colon tumors compared to tumor-free tissue of the distal colon [47] and CCL5 showing enhanced secretion in colon tumors whose gene expression profile indicated increased type-1 T cell activity [35]. Although CCL5 does not seem to mediate direct chemotactic effects on CD8^+^ T cells, its absence in orthotopic CRC tumor models interestingly resulted in the marked accumulation of intratumoral effector CD8^+^ T cells and in significantly reduced tumor growth [52]. These in-vivo-acquired data implicated a tumor-promoting role of CCL5, which can, at least partly, be explained by its capacity to positively influence the secretion of the calcium-binding protein S100a9 by tumor-associated macrophages (TAMs) [52], and thereby to suppress T cell activation and proliferation [53]. Moreover, CCL5 could be identified as a potent promoter of intratumoral T_reg_ accumulation and activation, which also significantly enhances their ability to kill CD8^+^ T cells and to dampen the T cell-driven antitumor immune response [51]. The concept that the colon tumor-promoting effect of CCL5 crucially depends on the involvement of the adaptive immune system was further supported by the observation that the inoculation of CCL5-deficient colon tumor cells into immunocompetent recipient mice, but not into B and T cell-lacking Rag1-deficient animals, resulted in impaired tumor growth [51]. Similar to CCL5, CCL3 has also been linked to increased colon tumor development, although, at least to our knowledge, no relevant impact of this CCR5-binding chemokine on the T cell-mediated control of colon cancer has been described so far. Obviously, it is more the potency of CCL3 to trigger the colonic accumulation of cancer-associated fibroblast and the subsequent production of fibroblast-derived growth factors that underlies its pro-tumorigenic character [54]. Regarding the ability of CCL3 to promote the recruitment of fibroblasts, it should be mentioned that cancer-associated fibroblasts represent an important source for the chemokine CXCL12, which has also been linked to an immunosuppressive and thus tumor-promoting microenvironment in the context of CRC and whose receptor CXCR4 showed increased expression levels in the tumor tissue of CRC patients with poor clinical prognosis [29,55,56]. Overall, the described involvement of the CCR5 ligands CCL5 and CCL3 in CRC-driven immune escape mechanisms and their tumor-promoting properties seemed to outweigh the capacity of CCL4 to trigger the recruitment of peripheral effector T cells in CRC metastases via CCR5 engagement [57]. Accordingly, the therapeutic use of the CCR5 inhibitor maraviroc in patients with advanced-stage metastatic colorectal cancer resulted in the marked induction of tumor cell death and decreased tumor cell proliferation within metastatic lesions, while the number of infiltrating CD8^+^ T cells detected in the invasive margin of liver metastasis remained mostly stable [57]. 

### 2.5. CCR6

Also of particular interest in the context of metastatic CRC disease, elevated levels of the chemokine receptor CCR6 in primary CRC lesions were found to be associated with the presence of liver metastases [58]. In accordance with these data, another study demonstrated that increased expression of the CCR6 ligand CCL20 in colon tumor tissue was associated with worse overall survival of CRC patients under neoadjuvant chemotherapy [27]. CCL20 is produced by colon cancer cells and tumor-associated macrophages and its release into the tumor microenvironment can even be intensified by the standard chemotherapeutic agent 5-fluorouracil [27,59]. As the suggested key mechanism underlying the enhancing influence of CCL20/CCR6 on CRC progression and chemoresistance, CCL20 is able to trigger the intratumoral accumulation of CCR6^+^ T_reg_ cells and, thereby, to counteract the antitumor immune response [27,59]. In some analogy to the enhanced CCL20-driven T_reg_ recruitment during neoadjuvant chemotherapy, surgical interventions might also bear the risk of promoting local T_reg_ accumulation in CRC and thereby tumor progression, with CCL18 discussed as the responsible chemokine [60].

## 3. Cytokines Influencing the Efficacy of the Antitumor Immune Response

**Figure 2:**
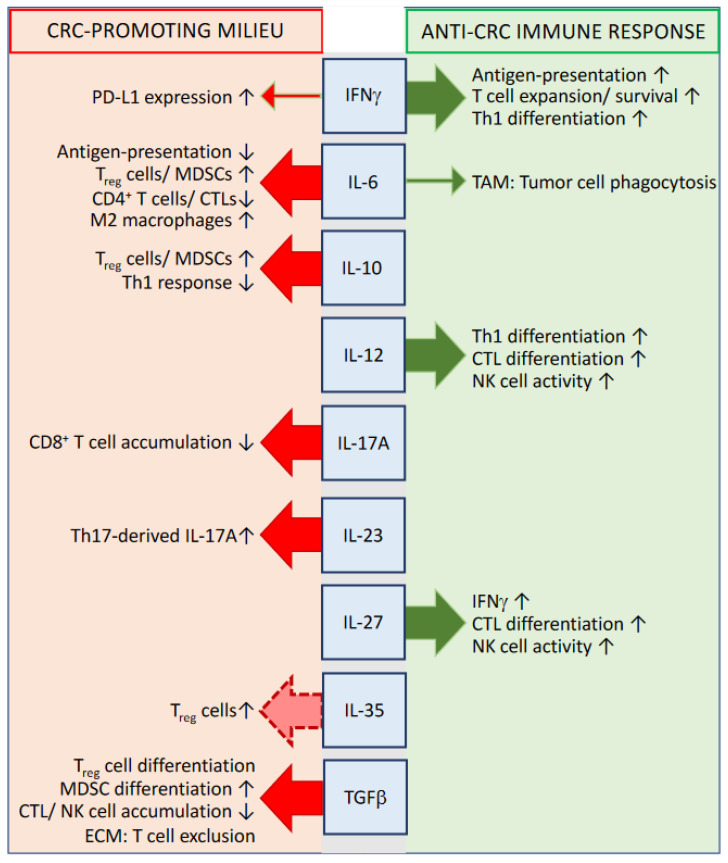
Cytokines of described relevance for the immune system-mediated control of CRC. Abbreviations: PD-L1 = programmed death ligand 1; TAM = tumor-associated macrophages; CTL = cytotoxic T lymphocytes; MDSC = myeloid-derived suppressor cell; ECM = extracellular matrix; ↑ = increased; ↓ = decreased.

While the primary role of chemokines is to induce and coordinate the recruitment of immune cells into the tumor microenvironment, cytokines serve predominantly as modulators of the activation, differentiation, and function of tumor-infiltrating immune cells [13,61] (Figure 2). Although a plethora of different cell types within the intestinal tumor tissue—such as, for instance, innate immune cells, cancer-associated fibroblasts, and dysplastic epithelial cells—relevantly contribute to and/or depend on the local cytokine network, tumor-infiltrating lymphocytes seem to function as the main actors in this context [3,13]. Locally accumulated CD4^+^ and CD8^+^ T cells do not only represent an important source for the release of pro- and anti-inflammatory cytokines into the tumor microenvironment, but are also key targets of intratumoral cytokine signaling. Dependent on their differentiation into distinct T helper cell subsets, T lymphocytes can either function as potent promoters of the antitumor immune response (e.g., Th1 cells and cytotoxic T lymphocytes (CTLs)) or support the further progression of tumor development (e.g., Th17 cells, Th22 cells, and T_reg_ cells) [13]. 

### 3.1. IFNγ

It is well established that the pro-inflammatory cytokine IFNγ mediates potent antitumor effects. In addition to its direct anti-proliferative impact on colon tumor cells [62] and its capacity to inhibit tumor-associated angiogenesis [63], IFNγ particularly supports the immune cell-driven tumor defense, including the MHC-I- and MHC-II-dependent presentation of tumor antigens and the activation, expansion, and survival of tumor-infiltrating effector T cells [13,64]. Accordingly, the presence of Th1 cells, NK cells, and CD8^+^ T cells, as the main cellular producers of the pro-inflammatory cytokine IFNγ, in colon tumors correlates with improved clinical prognosis [5,65,66], while downregulation of the IFNR1 chain of type I interferon on the protein level in the cancer cell compartment or the stromal cell compartment of CRC tumors was found to be associated with poor disease prognosis [67]. Interestingly, the microenvironmentally induced degradation of the IFNR1 protein in tumor-associated stromal cells and particularly in CD8^+^ T cells could recently be identified as a common process during CRC development, which is required for efficient tumor growth [67]. Analyses in the murine MC38 colon cancer model clearly indicated that the inhibition of the local IFN signaling via degradation of IFNAR1 resulted in the significantly decreased survival of tumor-infiltrating CD8^+^ T cells as the key cytolytic mediators of the immunological tumor control, and in the subsequent downregulation of cytolytic effector genes such as granzyme B and IFNγ [67]. Moreover, experimental data from the same study strongly suggested that the targeted stabilization of IFNR1 expression in colon tumors might improve the efficacy of anti-PD-1 therapy [67].

Despite the traditionally assumed and well-validated antitumor capacity of IFNγ, there is also growing evidence for a more ambiguous role of this cytokine in the fine-tuning of CD8^+^ T cell-driven CRC defense. In particular, the ability of IFNγ to promote the expression of PD ligand 1 (PD-L1) in cancer cells is considered evidence that it also has immunosuppressive properties. Indeed, the intratumoral expression of PD-L1 in a cohort of 181 CRC patients was found to be positively associated with the mRNA levels of IFNG and its downstream signaling molecules JAK2 and STAT1, and, on a functional level, IFNγ was able to induce enhanced PD-L1 expression in human colorectal cancer cells, thus strongly suggesting its supportive impact on the tumor cell-driven initiation of immune escape mechanisms [68]. In this context, it is noteworthy that a recent study pointed to the histone methyltransferase WHSC1 as an essential mediator of the IFNγ-promoted MHC-I expression in colon cancer cells, whose capacity to epigenetically modulate gene transcription did not seem to be involved in the IFNγ-induced expression of PD-L1. The identification of strategies to promote the enzymatic activity of WHSC1 in colon tumors thus appeared as a promising strategy to trigger selectively the antitumor properties of IFNγ in colon tumors without enhancing its potential involvement in immunosuppressive processes [69]. 

### 3.2. IL-17A and IL-17F

Almost two thirds of primary sporadic CRCs were estimated to show increased expression of IL-17A. In general, the enhanced presence of this cytokine was found to be associated with worse disease prognosis and more severe dysplasia [70,71,72]. In line with this, its deficiency in the azoxymethane (AOM)/dextran sodium sulfate (DSS) model of colitis-associated cancer significantly reduced the intestinal tumor burden. In contrast, augmented experimental colon tumor induction has been described in IL-17F-deficient mice, suggesting an at least partially opposing role of these two IL-17 family members in the pathogenesis of colon cancer [72]. In addition to distinguishing between the different members of the IL-17 family, it is further essential to differentially consider their influence on the various pathogenetic aspects involved in the complex process of CRC development—for example, their effect on tumor cells, angiogenesis, or immunological tumor defense. Taking into account the thematic focus of our article, which is the modulation of antitumor immune responses, it should only be briefly mentioned here that for IL-17A, the ability to increase tumor cell growth and tumor-associated neoangiogenesis has been described in the context of CRC, whereas IL-17F seems to influence both processes in the opposite direction [72]. In addition to the prognostically nonbeneficial role of IL-17A in the local microenvironment of CRC, CRC patients could be characterized by an increased frequency of IL-17^+^ CD4^+^ T cells in the peripheral blood [73], and increased serum levels of IL-17A in patients with advanced-stage colon tumors have been linked to the reduced tumor infiltration of CD8^+^ T cells [74]. Of mechanistical relevance, advanced-stage CRC patients could also be characterized by the significantly decreased expression of the chemokine receptor CXCR3 on the surfaces of circulating CD8^+^ T cells, while the CXCR3 ligand CXCL10 turned out to be upregulated in the tumor tissue. Together with the described capacity of IL-17A to inhibit the expression of CXCR3 in CD8^+^ T cells via STAT3 signaling, the acquired results thus implicated that Th17 cell-released IL-17A is able to dampen the immunological control of CRC disease via the IL-17A/STAT3/CXCR3 axis [74]. Together with γδT cells, Th17 cells appeared as the predominant source of IL-17 in the micromilieu of colon tumors, where their development turned out to be particularly dependent on the transcription factor Batf [14,75,76]. As already indicated by the description of the partly very different effects of IL-17A and IL-17F on CRC pathogenesis, it is obviously far too simplistic to automatically conclude from the pro-tumorigenic effect of IL-17A that an increased presence of Th17 cells in tumor tissue might be an indicator of poor disease prognosis. Rather, CRC-infiltrating Th17 cells showed some Smad7-induced plasticity towards T-bet/RORγt double-positive T cells characterized by low IL-17A and increased IFNγ expression [77] and could in general be described as multifunctional modulators of the local antitumor immune response, which are able to secrete a broad spectrum of different cytokines and chemokines in addition to IL-17 and, thereby, to support also important mechanisms of tumor defense [75]. In accordance with the described double-edged role of Th17 cells in the context of CRC, the number of tumor-infiltrating IL-17^+^ cells did not allow the disease outcome to be predicted [75]. However, the increased intratumoral frequency of the specific fraction of IL-17^+^ γδT cells indeed seemed to be associated with clinical parameters such as enhanced tumor size, vascular invasion, and lymph node metastasis, thus indicating CRC invasiveness and progression [14]. As with Th17 cells, tumor-infiltrating γδT cells do not exclusively produce IL-17 but are also considered as an important source of cytokines such as IL-8, GM-CSF, and TNFα, enabling them, in particular, to recruit polymorphonuclear myeloid-derived suppressor cells [78] into the tumor tissue and, thus, to contribute significantly to the establishment of an immunosuppressive and tumor-promoting milieu [14].

### 3.3. IL-10

As one of the cytokines integrally involved in the formation of an immunosuppressive tumor micromilieu, IL-10 was found to be markedly upregulated on the mRNA level in CRC-infiltrating T_reg_ cells compared to colorectal polyps and control colon tissue derived from healthy volunteers, and increased serum levels of IL-10 could be linked to therapy failure, decreased survival, and post-surgical disease recurrence in CRC patients [79,80,81,82]. Besides T_reg_ cells, i.e., Th2 cells, monocytes, intestinal macrophages, and colon tumor cells are relevant sources of IL-10 in the context of CRC [80]. In accordance with its prognostically negative role, experimental silencing of IL-10 secretion in the local environment of MC38 tumors was able to inhibit tumor growth and to improve the efficacy of combined chemoimmunotherapy by reducing the local accumulation of myeloid-derived suppressor cells and T_reg_ cells and by triggering Th1-mediated antitumor immunity [83,84]. However, in the APCmin^−/+^ model of sporadic CRC, IL-10 seemed to be responsible for the capacity of adoptively transferred T_reg_ cells to inhibit intestinal tumor development and growth. It can thus be hypothesized that IL-10 mediates a growth/survival-supporting effect directly on intestinal tumor cells and/or dampens the activity of effector CD4^+^ T cells in tumor-promoting chronic inflammatory processes [85,86], whereby both effects would oppose the experimentally demonstrated suppressive influence of IL-10 on the antitumor immune response and its negative prognostic relevance in human CRC disease. Thus, further studies are needed to better decipher the distinct and partly contrary effects of IL-10 on the different aspects of intestinal tumor disease and possibly to be able to therapeutically target them separately in the future.

### 3.4. IL-6

Increased concentrations of IL-6 in the peripheral blood of CRC patients turned out to be associated with an advanced tumor stage, the development of metastasis, tumor recurrence, and reduced survival [87,88,89,90]. Accordingly, the significantly diminished growth of experimentally induced orthotopic or inflammation-associated colon tumors could be observed in IL-6-deficient mice compared to tumor development in IL-6-proficient control mice. Moreover, tumor growth in wild-type mice could successfully be inhibited by anti-IL6R treatment [91,92,93]. IL-6 can be secreted by a large panel of different immune and non-immune cells [94], whereby lamina propria CD4^+^ T cells and, in particular, PU.1-driven Th9 cells, dendritic cells, and tumor cells could be identified as the main producers of this cytokine in colon tumors [93,95]. Mechanistically, the binding of IL-6 to the IL-6 receptor (IL6R) and the subsequent association with the glycoprotein gp130 is known to initiate STAT3 signaling in immune cells and epithelial cells [93]. The suggested unbeneficial influence of IL-6 on the fate of CRC disease was mainly attributed to its inhibitory effects on T cell-mediated aspects of antitumor immunity, as well as to its proliferative and anti-apoptotic effects on colon tumor cells, which are mainly mediated via the soluble IL-6 receptor (sIL6R) and IL-6 trans-signaling [93,96]. Regarding its dampening influence on the antitumor immune response, it has been demonstrated that the absence of IL-6 in host mice in an orthotopic CT26 colon tumor model resulted in the increased expression of MHC class I molecules on the surfaces of tumor cells and in the intratumoral accumulation of mature dendritic cells, CD4^+^ helper T cells, and CD8^+^ cytolytic effector T cells [91]. Moreover, for the same colon tumor model, another study described the additional IL-6-triggered regulation of the number of tumor-infiltrating T_reg_ cells and myeloid-derived suppressor cells, whereby overexpression of the cytokine in tumor cells resulted in the increased detectability of these two immunosuppressive cell types [87]. Moreover, in the context of colitis-associated colon cancer, IL-6 is known to shift macrophage polarization towards tumor-promoting M2 macrophages [97]. However, for completeness, it should also be mentioned that despite its predominantly pro-tumorigenic function, IL-6 has been described as an important activator of the macrophage-mediated phagocytosis of colon tumor cells, driving the required cytoskeleton rearrangement [98]. Moreover, in general, there is not only a unilateral influence of IL-6 on the composition and activation of the CRC immune microenvironment, but IL-6-mediated signaling is conversely also controlled by components of the tumor micromilieu. For instance, TGFβ could be identified as a potent inhibitor of IL-6-mediated tumor-supportive effects [93], whereas Th9 cell-released IL-9 is apparently able to increase I-L6 and IL-9 production by intratumoral Th9 cells in an auto-amplifying loop [95]. 

The fact that, in parallel to the upregulation of MHC class I molecules in the IL-6-deficient host milieu, a concomitant increase in PD-L1 expression was observed on the surface of IL-6-proficient CT26 tumor cells [91], implied that the therapeutic blockade of IL-6 signaling can potentially improve the responsiveness of CRC patients to checkpoint inhibitor therapies. Notably, the quality of the IL-6-mediated regulation of PD-L1 expression in colon tumor cells obviously depends on the source of IL-6 production, as the significant induction of this immunoregulatory molecule on the tumor cell surface could be detected in a similar manner either after the overexpression of IL-6 in the tumor cell compartment [87], after antibody-mediated systemic IL-6 blockade [92], or in the absence of IL-6 in the surrounding non-tumor cells [91]. However, in vivo findings provided experimental evidence for the synergistic and beneficial therapeutic effects of combined IL-6/ PD-L1 blockade in the CT26 colon cancer model [87,91,92]. 

### 3.5. IL-12 Family of Cytokines

The IL-12 family of cytokines consists of IL-12, IL-23, IL-27, and IL-35, with IL-12, IL-23, and IL-27 being mainly produced by macrophages and dendritic cells, while IL-35 is predominantly secreted by regulatory T cells but can also be expressed by tumor cells [99,100,101]. All four family members have in common that they represent heterodimers consisting of an α chain and an αβ chain and that their particular receptors initiate the JAK-STAT signaling pathways, while they markedly differ with regard to their immunological function and show a unique heterodimeric combination of their subunits. IL-12 consists of a p35 and a p40 chain, IL-23 comprises the p19 and the p40 protein, IL-27 was defined as a heterodimer of p28 and EBI3, and, in the most recently identified family member, IL-35 EBI3 forms a complex with p35 [101,102]. 

IL-12 is well known as a classic mediator of antitumor immunity, mainly attributed to its capacity to promote Th1 cell and CD8^+^ T cell differentiation and NK cell activation and, subsequently, to enhance IFNγ secretion, which is required for the efficient presentation of tumor antigens, T cell recruitment to the tumor site, and the intratumoral survival and expansion of cytolytic effector T cells [103,104,105,106,107,108]. Accordingly, the experimentally induced local increase in IL-12 levels in the tumor microenvironment showed clear therapeutic efficacy in different in vivo models of CRC disease and colon cancer metastasis [101,105,106,109,110]. Interestingly, a successful therapeutic response to local IL-12 therapy in a model of hepatic colon cancer metastasis was associated with increased expression of the IL-15 receptor alpha (IL15Ra) in tumor-specific CD8^+^ T cells, indicating the significant relevance of IL-12 for IL-15-supported long-lasting memory CD8^+^ T cell responses [111]. In accordance with this link between IL-12 and IL-15, there also exists experimental evidence that some of the IL-12-mediated immunological antitumor effects can even be observed in the absence of IFNγ, but then require the presence of IL-15 [112]. Moreover, another study reported that mice with a good therapeutic response to combined cyclophosphamide plus IL-12 induction therapy in the orthotopic CT26 colon cancer model were characterized by significantly decreased numbers and immunosuppressive activity of systemic and tumor-infiltrating T_reg_ cells [113]. Despite promising preclinical data, clinical studies performed so far on the applicability of IL-12-based strategies for the treatment of CRC are relevantly limited by the high level of toxicity associated with the systemic delivery of the cytokine [114] and fail to report a significant therapeutic advantage [101,115]. As a promising approach to potentially overcome the limitations caused by the poor systemic tolerability of IL-12, a recent preclinical study described a marked beneficial effect of intratumoral IL-12p70 mRNA therapy on tumor growth and tumor regression in the MC38 colon tumor model. Indeed, the intratumoral delivery of lipid nanoparticle-encapsulated IL-12p70 mRNA resulted in the increased secretion of IL-12p70 protein by the colon tumor cells and, subsequently, resulted in a Th1 shift in the tumor microenvironment, triggering IFNγ -driven and CD8^+^ T cell-dependent antitumor immunity [116]. 

In a quite similar way as described for IL-12, overexpression of IL-27 in colon cancer cells was able to trigger local IFNγ secretion, NK cell-mediated antitumor effects, and T cell-mediated tumor-specific cytotoxicity [117]. In particular, in CD4^+^ T cells and NK cells, but also in CD8^+^ T cells, a marked mRNA expression of the alpha subunit of the IL-27 receptor alpha could be detected [101]. Although experimental overexpression of IL-27 in the micromilieu of colon cancer resulted in decreased tumor growth even in the absence of IL-12p40, the functional repertoire of IL-27 also includes effects that could only be observed as synergistic effects together with IL-12, such as, for instance, the enhancement of IFNγ production in CD8^+^ T cells [118]. Other aspects of the tumor-controlling capacity of IL-27, such as its ability to induce the proliferation of naïve CD8^+^ T cells or the differentiation of CTLs, seem to be independent of the presence of the transcription factor T-bet, which crucially controls the fate of Th1 cells and CTLs [118,119]. Most likely, IL-27 is able to compensate for the lack of T-bet during the process of CTL differentiation by its demonstrated capacity to induce the augmented expression of Eomes [118], representing an alternative T-box transcription factor of described prognostic relevance in the clinical context of CRC [120]. 

In contrast to the beneficial effects of IL-12 and IL-27 on the immune system-mediated control of CRC development, IL-23 was found to promote the immune evasion of colon tumor cells [101,121]. Indeed, IL-23p19-deficient animals turned out to be protected against the experimental induction of colon cancer [76,101,122]. In human CRC patients, IL-23p19 mRNA and protein expression levels were found to be significantly increased in the tumor tissue compared to adjacent tumor-free colon tissue [76,123], and individuals with elevated IL-23p19 mRNA expression showed lower disease-free survival rates [124]. Mechanistically, the early CRC-induced loss of local intestinal barrier integrity and the subsequent entry of microbial products into the tumor microenvironment relevantly trigger the activation of IL-23-producing intratumoral myeloid cells [125]. In general, antigen-presenting cell-derived or neutrophil-derived IL-23 [126] is known to be crucially involved in the differentiation, maintenance, survival, and IL-23-responsiveness of Th17 cells [76,127,128] and thereby contributes to increased levels of IL-17A in the tumor microenvironment [76], favoring an immunosuppressive and tumor-promoting milieu. Accordingly, the presence of intratumoral IL-17A^+^ IL-23R^+^ IL-6^+^ CD4^+^ T cells was described to be associated with the development of colitis-associated cancer [76]. 

Compared with IL-12, IL-23, and IL-27, relatively little is known so far about the exact functional influence of the most recently discovered IL-12 family member, IL-35, on CRC pathogenesis. In good accordance with the generally suggested immunosuppressive and tumor-promoting function of IL-35 [129,130], the overall increased presence of the two IL-35 subunits p35 and EBI3 in colon tumor tissue reached the most pronounced expression levels in advanced and poorly differentiated colon tumors [99]. Notably, serum levels of IL-35 in patients diagnosed with CRC disease positively correlated with the number of circulating FoxP3 T_reg_ cells in the peripheral blood [99], which may indicate, beyond the fact that T_reg_ cells are considered the main source of IL-35, that IL-35 is involved in the CRC-associated induction of peripheral T_reg_ cells and thus in the initiation of tumor immune escape mechanisms.

### 3.6. TGFβ

In the colon tumor microenvironment, the anti-inflammatory cytokine TGFβ turned out to be mainly produced by cancer-associated fibroblasts but also by tumor-infiltrating T_reg_ cells and tumor cells [93,131]. Its increased expression in primary colon tumor specimens correlated with metastatic disease and poor prognosis. It should nevertheless be mentioned, at this point, that the direct interplay between TGFβ and colon cancer cells can obviously mediate both tumor-promoting and tumor growth-arresting effects depending on the stage and type of disease, emphasizing the complexity of its involvement in the pathogenesis of CRC [132,133]. However, focusing on its impact on the immune system-mediated control of colon cancer and CRC metastasis, TGFβ has been definitively identified as a driver of tumor immune escape mechanisms, and its targeted inhibition in different preclinical in vivo models successfully dampened the development and metastasis of colon cancer and increased the efficacy of adoptive NK cell therapy [131,134]. Besides its immunosuppressive function, which includes the local induction of T_reg_ cell differentiation from naïve CD4^+^ T cells and the generation of monocytic myeloid-derived suppressor cells from CD14^+^ monocytes [135,136,137], TGFβ serves as a key regulator of extracellular matrix (ECM) homeostasis. Via its ECM-modulating capacity, TGFβ contributes to the phenomenon of tumor T cell exclusion, which means that the accessibility of the tumor tissue for invading immune cells can be blocked by the formation of a collagen-rich ECM barrier [138]. Indeed, genes induced by TGFβ in normal colonic fibroblasts were found to be upregulated in colon tumors of CRC patients with poor prognosis [139]. Moreover, on a functional level, it could be demonstrated, in the in vivo MC38 colon cancer model, that the therapeutic blockade of TGFβ resulted in the increased tumor infiltration of CD8^+^ T cells and NK cells [138]. Regarding the effect of TGFβ on colon tumor-infiltrating CD4^+^ T cells, the increased presence of TGFβ in sporadic colon tumors was obviously accompanied by the augmented presence of IL-17A^+^ IL-22^+^ CD4^+^ T cells. On a functional level, in vivo studies in colitis-associated cancer were able to confirm that TGFβ relevantly contributed to the intratumoral accumulation of IL-17A^+^ IL-22^+^ CD4^+^ T cells and thereby obviously triggered IL-22-dependent tumor-promoting effects [140,141]. Finally, results from the same preclinical models interestingly suggested that the tumor micromilieu is able to further increase the TGFβ responsiveness of colon tumor-infiltrating CD4^+^ T cells by suppressing their expression of the TGFβ signaling-regulator Smurf2, with the consecutive induction of TGFβ receptor type II levels [142]. In summary, TGFβ can thus be seen as a relevant antagonist of the immunological control of CRC disease, both by limiting the presence of cytolytic effector immune cells in the tumor and by promoting the T cell-mediated secretion of pro-tumorigenic cytokines. However, it should also be considered that the T cell-specific blockade of TGFβ-signaling in the AOM/DSS model of colitis-associated cancer resulted in enhanced tumor formation [93], which was obviously driven by the increased intratumoral presence of T cell-released IL-6 and its capacity to trigger the growth of dysplastic epithelial cells. 

## 4. Conclusions and Targets beyond Chemokines and Cytokines

The highlighted multifaceted influence of chemokines and cytokines on the tumor microenvironment and, in particular, on the recruitment, intratumoral accumulation, and activation of lymphocytes suggests their strong potential as therapeutic targets to specifically enhance the immunological control of tumor disease. In this context, however, it is important to carefully take into account that most of these secreted immunomodulators rarely act selectively on a single immune cell type, depending on the expression profile of their respective receptors, and often also have relevant direct effects on tumor cells or mediate systemic effects outside the tumor environment. Preclinically validated strategies to limit the systemic side effects of cytokine/chemokine-based treatment strategies in the therapy of solid tumors, for instance, include the intratumoral induction of non-secretable membrane-bound but still functionally active cytokines or the ex vivo exposure of effector immune cells to selected cytokines prior to adoptive cell therapy.

Moreover, cytokines and chemokines are of course not the only players and potential therapeutic targets within the colon tumor microenvironment that are able to modify the efficiency of immune cell recruitment into the intestinal tumor tissue and the locally induced antitumor immune response. In particular, tumor-associated neoangiogenesis, which is promoted by local hypoxia, the deprivation of nutrients, and the secretion of different growth factors, represents an important and complex process that significantly impacts the successful infiltration of immune cells from the circulating blood into the tumor tissue [143]. For instance, binding of Vascular Endothelial Growth Factor A (VEGF-A) to its receptor, VEGFR-2, represents a very relevant trigger for promoting angiogenesis and neovascularization in the context of colorectal cancer. However, this is again a highly complex scenario, as the newly formed vascular network induced under the influence of the intratumoral disbalance between pro- and anti-angiogenic factors often shows structural and functional abnormalities, resulting in insufficient tissue perfusion and the hampered delivery of chemokine-attracted effector T cells [144,145]. Moreover, VEGF-A has also been demonstrated to directly promote the proliferation of VEGFR-2-expressing regulatory T cells in colon tumor-bearing mice. Accordingly, the treatment of CRC patients with the clinically approved VEGF-A-targeting antibody Bevacizumab resulted in a significantly reduced frequency of regulatory T cells in the peripheral blood, and the in vivo administration of a fusion protein consisting of an anti-VEGFR2 antibody and IFNα successfully enhanced the accumulation of CD8^+^ T cells in a murine colorectal cancer xenograft model [145,146,147,148]. 

Besides the sensing of a chemokine-mediated recruitment signal and the accessibility of the tumor tissue ensured by existing blood vessels, the ability of circulating immune cells to migrate from the blood into the tumor tissue also crucially depends on their surface expression profile of tissue-specific homing receptors. As a surface molecule of key relevance for T cell gut homing, the integrin β7 as a heterodimer either with integrin α4 or integrin αE promotes the adhesion of T cells at the endothelial wall and their subsequent retention in the intestinal tissue, respectively [149]. Indeed, the functional and prognostic relevance of integrin β7-mediated T cell homing could also be confirmed for the pathogenesis of CRC disease, although different studies reported partly conflicting data on the quality of its involvement. The increased intratumoral presence of integrin β7-postive cells was found to be associated with enhanced immune cell-mediated cytolytic activity and the improved survival of CRC patients [150], while another study described that decreased expression levels of MAdCAM-1, the ligand of α4β7, in human colon cancer tissue pointed to an improved colon tumor prognosis [151]. In the orthotopic MC38 colon tumor model, mice carrying a genetic deficiency for integrin β7 showed the reduced intratumoral infiltration of activated T cells, B cells, NK cells, and DCs and developed larger tumors, overall implicating that integrin β7 is required for efficient antitumor immunity. However, besides these quite recent data, there also exist experimental data published by another group already in 2017 that functionally linked the absence of integrin β7 to the slower growth of sporadic colon tumors [151]. Irrespective of the fact that the exact clinical consequence and the underlying mechanisms of β7 integrin-mediated endothelial adhesion and the intratumoral accumulation of immune cells, as well as the possible involvement of other integrins and integrin receptors, certainly need to be investigated in more detail in future studies, anti-adhesion and anti-integrin therapies not only represent a valuable treatment approach in the therapy of chronic inflammatory diseases [152], but might also emerge as a promising and exciting therapeutic option for triggering the immunological tumor control in CRC disease.

In conclusion, we hope that, in this review article, we have succeeded in demonstrating the diversity and functional complexity but also the great therapeutic potential of the various factors involved in immune cell recruitment and local immune cell activation in the colon tumor microenvironment, which are highly critical for modulating the antitumor immune response, for determining the responsiveness to immunotherapy, and, finally, for improving CRC prognosis.
